# Delayed cystic fibrosis diagnosis due to presumed celiac disease-A case report from Syria

**DOI:** 10.1186/s12887-023-03982-7

**Published:** 2023-04-11

**Authors:** Yahia Ranjous, Abdulrahman Al Balkhi, Nazir Alahmad, Ali Asaad, Ayman Ali

**Affiliations:** 1 Faculty of Medicine, Damascus University, Damascus, Syria; 2 Gastroenterology Department, Faculty of Medicine, Damascus University, Damascus, Syria

**Keywords:** Cystic fibrosis, Celiac disease, Misdiagnosis

## Abstract

**Background:**

This case report describes a cystic fibrosis case after 7 years of a presumed diagnosis of celiac disease without confirming laboratory tests and biopsies. Both cystic fibrosis and celiac disease cause malnutrition, malabsorption, and failure to thrive. Also, the occurrence of cystic fibrosis in celiac disease patients is higher than in the normal population. Therefore, the differentiation between the two diseases might be challenging. This article highlights the reason for the confusion between cystic fibrosis and celiac disease and emphasizes the importance of not skipping the necessary investigations no matter how difficult it is to perform them.

**Case presentation:**

This report details the case history of a patient presumed to have celiac disease for 7 years without confirming investigations. He developed multiple respiratory infections and weight loss throughout the 7 years but was only diagnosed with cystic fibrosis after hospitalization for gradual abdominal distension and productive cough. Chest CT showed atelectasis in the right upper lobe, tree-in-bud sign on both sides, and right periumbilical mass with several enlargements in the mediastinal nodes. Ascites paracentesis revealed a high SAAG gradient and low-protein fluid. The sweat chloride test resulted in a chloride level of 90 mEq/L, which confirmed the cystic fibrosis diagnosis. Subsequent genetic testing revealed the rare G85E mutation.

**Conclusion:**

This report highlights the potential for diagnostic confusion between cystic fibrosis and celiac disease. Also, it reminds physicians about the importance of taking a detailed medical history and performing the essential investigations no matter how difficult it is to do them. Finally, it emphasizes the need to verify the patient’s previous medical history in case there is no official documentation of his case. This should be considered particularly in rural areas in low-income countries where the possibility of medical malpractice should not be forgotten.

## Background

Cystic Fibrosis (CF) is an autosomal recessive disease caused by a mutation in a gene on chromosome 7. This gene encodes a transmembrane protein called CFTR, which functions as a chloride channel [1]. CF is a multiorgan disease that manifests mainly with recurrent pulmonary infections, meconium ileus, malnutrition, and failure to thrive [2]. This disease is relatively rare in the middle east, and the approximate prevalence is about 1 in 30,000 to 1 in 50,000 [3], [4].

On the other hand, celiac disease (CD) is an autoimmune disorder that is triggered by gluten, a protein mainly found in wheat [5]. The main symptoms of CD are diarrhea and malabsorption [6]. Celiac disease prevalence in Syria is about 1.5% [7].

The two aforementioned diseases share similar features and were not distinguished from each other until 1938 [8]. It was noticed then on autopsy that some patients who were supposed to have celiac disease had pancreatic fibrosis [9]. Both CF and CD cause malnutrition, malabsorption, and failure to thrive. Also, the occurrence of CD in CF patients is 2–3 times higher than in the normal population [10]. Moreover, having CD might elevate sweat electrolytes, leading to a false positive CF diagnosis [11]. Therefore, distinguishing between CD and CF could be challenging. These difficulties are more notable in poor areas at times of crisis where necessary investigations are not always available. In this case report, we report a case of a 16-year-old male who was diagnosed with cystic fibrosis after 7 years of a false diagnosis of celiac disease without confirming laboratory tests and biopsies.

## Case presentation

A 16-year-old male was diagnosed with celiac disease 7 years ago. This was a clinical diagnosis based on clinical symptoms of steatorrhea and malnutrition. No serology or duodenal biopsy was performed due to the lack of resources at times of crisis. The patient was put on an experimental celiac disease gluten-free diet but he did not adhere to it. During these 7 years, steatorrhea persisted and the child had associated progressive weight loss. Also, he was repeatedly hospitalized for respiratory infections.

At the present time, he was referred to Assad University Hospital after a 2-week history of gradual abdominal distension and fever. He had a productive cough with yellow sputum for ten days and second-grade dyspnea. On the physical examination, the patient had pallor, clubbing, and pitting edema. He weighed 40 kg, his height was 150 cm, and with BMI = 17.7 Kg/m^2^. Abdominal examination showed generalized tenderness and shifting dullness. On the lung examination, there was generalized wheezing in the two lungs, prolonged exhalation, and dullness in the right apex pulmonis. Groin, chest, and armpit hair were absent, with Tanner stage = 1. Laboratory evaluation showed: hemoglobin 9.9 g/dL; total protein 5 g/dL; albumin 2.8 g/dL; ALT 81 IU/L; AST 98 IU/L; ALP 320 IU/L; total bilirubin 2 mg/dL; direct bilirubin 1.6 mg/dL; PT 31%; INR 2,4; Total IgA 580 mg/dL; TTG IgA negative. Chest X-ray revealed opacity in the right apex pulmonis with bilateral interstitial alveolar infiltrates. A computed tomography scan (CT) of the abdomen showed a moderate amount of fluid, severe heterogeneity of the hepatic tissue, and a small-sized pancreas with fatty infiltrations. On chest CT, we found atelectasis in the right upper lobe, tree-in-bud sign on both sides, and right periumbilical mass with several enlargements in the mediastinal nodes. Ascites paracentesis showed a high SAAG gradient and low-protein fluid. Esophagogastroduodenoscopy showed esophageal varices < 5 mm. Duodenal biopsies did not show atrophy or inflammation. While stomach biopsies showed mild chronic nonspecific inflammation. On bronchoscopy, there were massive purulent discharges that plug the right bronchus. Samples were taken to culture after suctioning the plugging discharges. TTG IgA negative result and negative findings in biopsies ruled out the former presumed celiac disease. The sweat chloride test resulted in a chloride level of 90 mEq/L, which confirmed the CF diagnosis. Steatorrhea improvement with pancreatic enzyme replacement therapy and the small-sized pancreas confirmed exocrine pancreatic insufficiency. Subsequent genetic testing revealed the rare G85E mutation. The patient’s health improved with treatment and he was discharged after recovery. Later, he was hospitalized again because of another pulmonary infection, resulting in respiratory failure and death.

## Discussion and conclusions

The differentiation between CF and CD can be challenging for many reasons. Firstly, both CD and CF cause malnutrition. Secondly, there is a coexistence possibility of the two diseases [10]. Finally, CD can elevate sweat electrolytes mimicking CF [11]. However, with a precise medical history and investigations, the final diagnosis can be made.

Total IgA and IgA tissue transglutaminase (tTG) should be tested in the serum to diagnose a patient with suspected CD. If the result is positive, then duodenal biopsies are necessary to confirm the diagnosis [12]. Also, HLA genetic testing can be useful in some rare specific cases [13], [14].

On the other hand, the first investigation in suspected CF patients is the sweat chloride test. If the test result is $\ge 60 mmol/L$, then the diagnosis is confirmed. And if it is $\le 29 mmol/L$, CF diagnosis is excluded. However, further study is required if the result is indecisive between $30 \text{a}\text{n}\text{d} 59 mmol/L$ [15].

The coexistence of the two diseases is well-known, and many studies showed a higher prevalence of CD in CF patients [16], [17], [18]. Imrei, M. et al. showed that the CD prevalence in CF patients is about two times higher than in the general population. Therefore, they recommended routine screening for CD in CF patients [19].

The first investigation in suspected CF patients is the sweat chloride test. Thus, many cases that cause abnormality in sweat electrolytes, such as CD, could be diagnosed by mistake as CF [11], [20], [21]. If it is difficult to screen CF patients for CD because of a lack of resources, doctors should at least keep in mind the coexistence of CD and CF and the possibility of a false positive sweat chloride test. Thus, any CF patient whose malnutrition does not get better with CF treatment will be tested for CD and other potential causes [16], [22].

Many mutations can affect the CFTR gene and cause cystic fibrosis [1]. Jarjour RA et al. study about CF mutations prevalence in Syria has shown that the G85E mutation in our patient is relatively rare, with only 4% prevalence in CF patients [23]. This missense mutation causes a replacement of glycine by glutamic acid at amino acid 85 [24]. It is classified as a class II mutation. This reduces CFTR molecules reaching the cell surface because of premature degradation by the endoplasmic reticulum [25]. Clinically, patients with G85E mutation have a more severe phenotype. They have a higher prevalence of pancreatic insufficiency and liver cirrhosis, worse BMI, and more frequent failure to thrive at diagnosis [26].

Our case differs from the previous literature in the absence of CD despite a false diagnosis with it based on clinical features without confirming serum tests and biopsy. The uncommon cystic fibrosis disease which had all the typical clinical features from the beginning was wrongly labeled something else. And finally, when the diagnosis was made, it was too late for the child. The reason for this lack of specialist care could be partially attributed to the war and crisis in Syria that made it difficult to get medical healthcare, especially in rural areas.

In conclusion, this case reminds us of the importance of taking a detailed medical history and checking previous investigations that the patient has done. Essential investigations should not be skipped no matter how difficult it is to do them. Also, it emphasizes the importance of verifying the previous diagnoses that the patient says he has if there were no confirming investigations or official documentation of his previous admittances to hospitals. This should be considered particularly in times of war and crisis. Finally, in places where CD is not routinely screened in CF patients, it should be kept in mind if the patient does not respond properly to CF treatment.

## Data Availability

The laboratory tests and imaging results are available from the corresponding author on reasonable request.

## List of Abbreviations

CF: Cystic fibrosis
CD: Celiac disease
BMI: Body mass index
